# Interprofessional Collaboration and Exploratory Implementation Support for Community Pharmacy Influenza Vaccination in Northwestern Romania: A Cross-Sectional Survey of Pharmacists and Family Physicians

**DOI:** 10.3390/epidemiologia7050132

**Published:** 2026-09-22

**Authors:** Adina Honoria Sabadoș (Budea), Daniela Rahotă, Teodor Traian Maghiar, Timea Claudia Ghitea, Laura Gratiela Vicaș, Lucia Georgeta Daina

**Affiliations:** 1 Doctoral School of Biological and Biomedical Sciences, University of Oradea, 1 University Street, 410087 Oradea, Romania; sabados.adinahonoria@student.uoradea.ro; 2 Department of Morphological Disciplines, Faculty of Medicine and Pharmacy, University of Oradea, 410028 Oradea, Romania; dr.rahota@gmail.com (D.R.); uro_doruletul@yahoo.com (T.T.M.); 3 Pharmacy Department, Faculty of Medicine and Pharmacy, University of Oradea, 1 University Street, 410087 Oradea, Romania; laura.vicas@gmail.com; 4 Department of Psycho-Neurosciences and Recovery, Faculty of Medicine and Pharmacy, University of Oradea, 1 December Sq., 410081 Oradea, Romania; lucidaina@gmail.com

**Keywords:** implementation support, community pharmacy, influenza vaccination, pharmacists, family physicians, interprofessional collaboration, cross-sectional survey, exploratory score, psychometric assessment, sensitivity analysis

## Abstract

Background: Romania introduced pharmacist-administered influenza vaccination in community pharmacies in 2022, but implementation depends on professional support, workflow capacity, and interprofessional coordination. Because the present questionnaire did not measure the full organisational construct of implementation readiness, we developed an exploratory, study-specific implementation-support score (IRS) from three attitudinal items. Methods: We conducted a cross-sectional survey in Northwestern Romania between 1 October 2025 and 30 April 2026. Of 761 questionnaires distributed, 700 were returned; 22 completely blank questionnaires were excluded, leaving 678 analysable responses (340 pharmacists and 338 family physicians). The IRS was the mean of perceived contribution to vaccination coverage, the pharmacist’s role in the multidisciplinary team, and willingness to recommend pharmacy-based vaccination. Internal coherence was assessed in the development sample. Associations with IRS ≥ 4 were examined using multivariable logistic regression, and a heteroskedasticity-robust linear regression using the continuous IRS was prespecified as a sensitivity analysis. Results: The overall IRS median was 3.00 (IQR 2.33–4.00); the mean was 3.09 ± 1.10. Pharmacists had higher scores than family physicians (median 3.67 vs. 2.67; mean 3.47 ± 1.02 vs. 2.70 ± 1.03; Mann–Whitney U = 81,200.5, *p* < 0.001). An IRS ≥ 4 was observed in 185 participants (27.3%), including 136 pharmacists (40.0%) and 49 family physicians (14.5%). In the adjusted logistic model, pharmacist status (aOR 4.41, 95% CI 2.66–7.33), considering direct collaboration absolutely necessary (aOR 4.22, 95% CI 1.42–12.52), annual influenza vaccination (aOR 2.98, 95% CI 1.56–5.69), and awareness of pharmacy vaccination (aOR 2.06, 95% CI 1.10–3.85) were associated with IRS ≥ 4. The continuous-score sensitivity analysis showed similar associations: pharmacist status (β 0.68, 95% CI 0.49–0.87), direct collaboration absolutely necessary (β 1.17, 95% CI 0.85–1.49), annual vaccination (β 0.51, 95% CI 0.28–0.74), and awareness (β 0.24, 95% CI 0.02–0.46). Conclusions: The three-item IRS showed internal coherence in this sample but represents proximal professional support rather than a validated measure of organisational implementation readiness. The findings are exploratory and associative; they should not be used as a validated institutional or policy decision threshold.

## 1. Introduction

Seasonal influenza continues to represent a major public health burden despite the widespread availability of safe and effective vaccines. Annual influenza vaccination remains the most effective preventive strategy for reducing influenza-related morbidity, mortality, and healthcare utilisation, particularly among vulnerable populations such as older adults, patients with chronic diseases, pregnant women, and immunocompromised individuals. Because healthcare professionals play a central role in recommending and administering vaccines, their attitudes toward vaccination services directly influence vaccine uptake and public confidence in immunisation programmes [1,2].

In recent years, many healthcare systems have expanded the role of community pharmacists by authorising pharmacist-administered influenza vaccination. This model has been associated with improved accessibility, extended service availability, reduced waiting times, and increased vaccination coverage, particularly among individuals who encounter barriers to accessing traditional primary care services. Community pharmacies provide one of the most accessible healthcare settings and have therefore become increasingly important partners in preventive medicine [3,4].

Although legislative approval represents an essential prerequisite for pharmacist-administered vaccination, successful implementation depends on considerably more than regulatory change. Implementation science emphasises that introducing a new healthcare service requires organisational preparedness, professional engagement, and effective collaboration between all stakeholders involved in patient care. Consequently, healthcare professionals’ readiness to support implementation has emerged as a critical determinant of whether newly authorised services become successfully integrated into routine clinical practice [5,6].

Interprofessional collaboration is particularly relevant in the context of pharmacy-based influenza vaccination because pharmacists and family physicians perform complementary rather than competing roles within vaccination programmes. Family physicians remain responsible for comprehensive patient management, while pharmacists contribute through increased accessibility, patient education, vaccine administration, and opportunistic vaccination. Effective implementation therefore requires clear communication pathways, mutual professional trust, shared clinical responsibilities, and well-defined organisational procedures [7,8].

Previous research has primarily investigated vaccine acceptance, pharmacists’ willingness to administer vaccines, or patients’ preferences regarding vaccination settings. Comparatively few studies have examined healthcare professionals’ preparedness to implement pharmacist-administered vaccination as a coordinated multidisciplinary service. Furthermore, implementation readiness is unlikely to depend on a single attitude. Perceived benefits for vaccination coverage, recognition of pharmacists’ professional role, willingness to recommend the service under appropriate regulatory conditions, awareness of pharmacy-based vaccination, and confidence in interprofessional collaboration may jointly determine whether healthcare professionals support implementation [9,10].

Implementation readiness is a multilevel construct that may include individual commitment, organisational capability, leadership, implementation climate, resources, workflow preparedness, and operational feasibility. The present questionnaire measured only a limited subset of these dimensions. Accordingly, the three-item composite is described here as an exploratory implementation-support score (IRS), and its results are interpreted as professional attitudes that may be relevant to implementation rather than as a direct measure of organisational readiness.

Romania is a relevant setting because pharmacist-administered influenza vaccination was introduced into community pharmacies in 2022 under Ministry of Health Order no. 3262/19 October 2022. Pharmacists providing the service are required to complete accredited training, and subsequent reimbursement provisions created 50% reimbursement for some adults and 100% reimbursement for several priority groups. National evaluations have reported that the authorised service remains concentrated mainly in urban pharmacies and that reimbursement, staffing, logistics, and remuneration remain practical implementation issues [6,11,12,13]. The present study did not collect pharmacy-level urban/rural status, authorisation, training, reimbursement, or staffing data; these omissions limit inferences about organisational readiness.

Therefore, the primary objective was to construct and describe a preliminary, study-specific implementation-support score (IRS) from three attitudinal items and to assess its internal coherence and dimensional structure in the development sample. A secondary objective was to explore demographic, professional, behavioural, and collaborative correlates of IRS ≥ 4 and to test whether the main associations were similar when IRS was analysed as a continuous outcome. No claim of external validation, measurement invariance, criterion validity, or a general organisational cut-off was intended.

We hypothesised that IRS values would differ by professional category and would be associated with awareness of pharmacy-based vaccination, personal influenza vaccination behaviour, and perceived need for interprofessional collaboration. Because the design was cross-sectional, all hypotheses were evaluated as associations rather than causal effects.

## 2. Materials and Methods

### 2.1. Study Design and Participants

This quantitative cross-sectional questionnaire study was conducted among pharmacists and family physicians practising in Northwestern Romania between 1 October 2025 and 30 April 2026. Recruitment covered Bihor, Cluj, Satu Mare, Maramureș, Bistrița-Năsăud, and Sălaj counties.

Participants were approached through participating community pharmacies using consecutive non-probability sampling. The analysis file contained 372 respondents from Bihor, 114 from Cluj, 67 from Satu Mare, 47 from Maramureș, 45 from Bistrița-Năsăud, and 33 from Sălaj. The number of participating pharmacies, pharmacy-level selection procedures, urban/rural status, and site-level clustering were not recorded in the supplied database. Family physicians recruited through pharmacy-linked sites may therefore be more likely to have existing contact with pharmacists than the wider physician population.

Eligible participants were pharmacists or family physicians aged ≥18 years who held a valid professional licence, were professionally active in one of the six included counties, provided informed consent, and submitted a usable questionnaire. Individuals were excluded if they were younger than 18 years, did not hold a valid professional licence, were not professionally active, did not provide informed consent, or returned a completely blank questionnaire.

According to the field distribution log, 761 paper questionnaires were handed out, and 700 were returned (overall return rate 92.0%). The log did not preserve profession-specific denominators, so separate response rates for pharmacists and family physicians could not be calculated. Twenty-two returned questionnaires were completely blank and were excluded, leaving 678 analysable questionnaires. Thus, usable questionnaires represented 89.1% of all distributed questionnaires and 96.9% of returned questionnaires. The final sample comprised 340 pharmacists (50.1%) and 338 family physicians (49.9%).

### 2.2. Questionnaire

The questionnaire was administered in Romanian and contained demographic questions and closed-response items addressing influenza vaccination, pharmacy-based vaccination, and physician–pharmacist collaboration. The present article analyses only the quantitative closed-response data. The complete verified Romanian questionnaire, an author-approved English translation, all response anchors, and the corresponding coding instructions are reported in Supplementary File S1.

The questionnaire was drafted from the published literature and revised within the research team for clarity and relevance to the Romanian healthcare context. No formal external expert review, structured content-validity assessment, pilot testing, cognitive interviewing, or formal pretesting was documented. This limitation is important because the three-item IRS was constructed retrospectively from an instrument that was not developed as a validated psychometric scale.

For reproducibility, the author-approved English translations of the three items used to calculate the IRS are reproduced below. The original Romanian wording, all response anchors, and complete coding instructions are retained in Supplementary File S1. The items represent perceived contribution to vaccination coverage, the pharmacist’s role within the multidisciplinary team, and willingness to recommend pharmacy-based vaccination.

Item 1 (database variable @34): “To what extent do you consider that administering influenza vaccines in community pharmacies could contribute to increasing influenza vaccination coverage?”

Item 2 (database variable @36): “How important do you consider the pharmacist’s role within the multidisciplinary team involved in influenza vaccination?”

Item 3 (database variable @40): “To what extent would you be willing to recommend community pharmacy-based influenza vaccination to patients?”

Each item was scored on a five-point response scale, with higher scores indicating greater support for pharmacy-based influenza vaccination. Items 1 and 3 used the anchors 1 = not at all, 2 = to a small extent, 3 = to a moderate extent, 4 = to a great extent, and 5 = to a very great extent. Item 2 used 1 = not at all important, 2 = slightly important, 3 = moderately important, 4 = very important, and 5 = extremely important.

No formal pilot testing, cognitive interviewing, pretesting, or structured content-validity study was documented before field administration. Therefore, both the questionnaire and the retrospectively constructed IRS are treated as exploratory and study-specific rather than as formally validated measurement instruments.

### 2.3. Development of the Exploratory Implementation-Support Score

The IRS was constructed retrospectively from three existing questionnaire items selected on conceptual grounds because they represented proximal attitudinal support for implementation: perceived population benefit, recognition of the pharmacist’s multidisciplinary role, and willingness to recommend the service. These items do not measure organisational resources, leadership, workflow capacity, implementation climate, reimbursement, or operational feasibility. For this reason, the score is referred to as an exploratory implementation-support score, while the abbreviation IRS is retained for continuity with the original analysis.

The three source variables were @34, @36, and @40 in the supplied database:

- @34: perceived contribution of pharmacy influenza vaccination to increasing vaccination coverage;
- @36: perceived importance of the pharmacist’s role within the multidisciplinary vaccination team;
- @40: willingness to recommend pharmacy-based influenza vaccination to patients.

Each source variable was coded from 1 to 5 in the supplied dataset, with higher values interpreted as greater support. The score was calculated as the arithmetic mean of the three items, preserving a theoretical range of 1–5. All 678 nonblank records had complete values for the three components.

The IRS was calculated as the arithmetic mean of the three items, preserving a possible range of 1–5. It was computed only when all three component responses were available; all 678 nonblank records met this requirement.

Internal coherence was assessed with pairwise Spearman correlations and Cronbach’s alpha. KMO and Bartlett’s test were calculated from the item-correlation matrix, and PCA was performed on the standardised-item correlation matrix. These analyses describe internal structure in the development sample only; they do not establish construct validity, criterion validity, temporal stability, measurement invariance, responsiveness, or external validity.

For the primary exploratory logistic analysis, IRS ≥ 4 was used as a descriptive threshold corresponding to an average response of at least 4 across the three items. This threshold was not derived from criterion validation. The continuous IRS was analysed in a prespecified sensitivity model using heteroskedasticity-robust linear regression with the same covariates.

### 2.4. Statistical Analysis

Likert outcomes were summarised primarily with medians and IQRs; means and SDs were retained as supplementary descriptive information. Categorical variables were presented as frequencies and percentages. Mann–Whitney U tests were used for ordinal outcomes and Pearson chi-square tests for categorical outcomes.

Comparisons between pharmacists and family physicians were performed using the Mann–Whitney U test for ordinal variables and the Pearson chi-square test for categorical variables.

Within-sample IRS assessment used Cronbach’s alpha, Spearman item correlations, KMO, Bartlett’s test of sphericity, and PCA of the standardised-item correlation matrix. Reported PCA loadings were correlations between each standardised item and the first principal component.

Independent associations with IRS ≥ 4 were investigated using multivariable binary logistic regression. The prespecified covariates were professional category, current physician–pharmacist collaboration, perceived need for direct collaboration, awareness of pharmacy-based vaccination, personal influenza vaccination behaviour, age group, sex, and professional experience.

The dependent variable was IRS ≥ 4, used as an exploratory dichotomisation rather than as a validated cut-off.

Independent variables entered into the model included the following:

- Professional category;
- Perceived effectiveness of current physician–pharmacist collaboration;
- Perceived need for direct interprofessional collaboration;
- Awareness of pharmacy-based vaccination services;
- Personal influenza vaccination behaviour;
- Age group;
- Sex;
- Professional experience.

The model contained 18 explanatory parameters plus an intercept (19 estimated coefficients in total). Reference categories were family physician, ineffective current collaboration, direct collaboration not necessary, unaware of pharmacy vaccination, never vaccinated against influenza, age ≥55 years, male sex, and >20 years of professional experience. Crude and adjusted odds ratios with 95% CIs were calculated. Multicollinearity was assessed using variance inflation factors (VIFs), and influential observations were screened using Cook’s distance. No interaction terms were examined because the analysis was exploratory, and the model specification was prespecified.

Overall fit was summarised with the likelihood-ratio test, Nagelkerke’s pseudo-R^2^, the area under the receiver operating characteristic curve (AUC), and the Hosmer–Lemeshow test. Nagelkerke’s pseudo-R^2^ is reported as a model-fit index and is not interpreted as literal variance explained in the ordinary linear-regression sense.

No adjustment for multiple comparisons was applied; the item-level group comparisons and regression analyses were prespecified as exploratory. A two-sided *p*-value < 0.05 was considered statistically significant.

All statistical analyses were performed using Python (version 3.12).

### 2.5. Data Quality

Before analysis, equivalent text variants were harmonised, including “Medic familie” with “Medic de familie”, Romanian hyphen/en-dash variants, and equivalent direct-collaboration responses. The current-collaboration variable was coded as 0 = not applicable, 1 = ineffective, 2 = partial, and 3 = effective. The original CSV was not overwritten.

All 678 nonblank records had complete values for IRS variables @34, @36, and @40, and were retained for the within-sample IRS assessment and the reported multivariable model.

### 2.6. Ethical Considerations

The study was conducted in accordance with the ethical principles of the Declaration of Helsinki.

Participation was voluntary and anonymous, and no personally identifiable information was collected. All participants provided informed consent before completing the questionnaire.

The study protocol received approval from the appropriate institutional ethics committee of University of Oradea (approval number 37; approval date 30 September 2025).

## 3. Results

### 3.1. Sample Characteristics and Descriptive Findings

The analysis included 678 complete, nonblank records after exclusion of 22 blank rows. Respondents were distributed across six counties: Bihor 372 (54.9%), Cluj 114 (16.8%), Satu Mare 67 (9.9%), Maramureș 47 (6.9%), Bistrița-Năsăud 45 (6.6%), and Sălaj 33 (4.9%). The dataset did not contain pharmacy identifiers or urban/rural status, so pharmacy-level representativeness and clustering could not be assessed.

Direct physician–pharmacist collaboration was considered absolutely necessary by 208 pharmacists (61.2%) and 183 family physicians (54.1%); it was considered necessary in selected cases by 106 pharmacists (31.2%) and 102 family physicians (30.2%). Awareness that influenza vaccination could be provided in community pharmacies was reported by 566 participants (83.5%). Annual personal influenza vaccination was reported by 354 participants (52.2%), occasional vaccination by 235 (34.7%), and no previous influenza vaccination by 89 (13.1%).

Perceived current collaboration was coded as effective for 171 respondents (25.2%), partial for 268 (39.5%), ineffective for 215 (31.7%), and not applicable for 24 (3.5%). These distributions indicate substantial professional interest in collaboration but also heterogeneous experience of current interprofessional practice.

The remaining questionnaire outcomes are reported in Table 1 and the corresponding figures (Figure 1). Because many item-level comparisons were exploratory and partially overlapping, Section 3 focuses on the IRS components and the prespecified predictors rather than repeating every questionnaire distribution.

### 3.2. Differences Between Pharmacists and Family Physicians

Pharmacists reported more favourable distributions than family physicians for all three IRS components. Median coverage-benefit scores were 3 (IQR 2–4) versus 2 (IQR 2–3), median pharmacist-role scores were 4 (IQR 3–5) versus 3 (IQR 2–4), and median willingness-to-recommend scores were 4 (IQR 3–5) versus 3 (IQR 1–4), respectively (all Mann–Whitney *p* < 0.001). Means are shown in Table 1 as supplementary descriptive information.

The item concerning whether pharmacy vaccination should be restricted to exceptional circumstances was analysed separately because higher scores indicate stronger support for restriction. Pharmacists had higher scores than family physicians (median 3 [IQR 2–5] vs. 2 [IQR 1–4], *p* < 0.001); this item was not included in the IRS.

The distribution of current collaboration differed by profession (χ^2^ = 29.73, df = 3, *p* < 0.001). Effective collaboration was reported by 140 pharmacists (41.2%) and 75 family physicians (22.2%); partial collaboration by 120 (35.3%) and 148 (43.8%); ineffective collaboration by 68 (20.0%) and 103 (30.5%); and not applicable by 12 (3.5%) and 12 (3.6%), respectively.

The distribution of perceived need for direct collaboration also differed (χ^2^ = 12.95, df = 3, *p* = 0.005), although a majority in both groups considered direct collaboration absolutely necessary.

Perceived patient preference for receiving influenza vaccination from the family physician was reported by 303 pharmacists (89.1%) and 329 family physicians (97.3%). The difference in perceived preferred location was statistically significant (χ^2^ = 26.63, df = 3, *p* < 0.001).

The distribution of perceived patient requests for pharmacy vaccination differed between professions (χ^2^ = 143.87, df = 4, *p* < 0.001). These item-level findings are exploratory and are not interpreted as evidence of causal effects or general population preferences.

Overall, pharmacists expressed stronger support on the three IRS items, whereas family physicians more often reported partial or ineffective current collaboration. Detailed medians, IQRs, means, and Mann–Whitney statistics are provided in Table 1.

### 3.3. Exploratory Development and Within-Sample Assessment of the IRS

The exploratory IRS was calculated as the mean of the three attitudinal items concerning coverage benefit, the pharmacist’s multidisciplinary role, and willingness to recommend pharmacy vaccination. The selection was conceptual rather than statistically optimised.

All 678 respondents had complete values for the three components. The overall IRS median was 3.00 (IQR 2.33–4.00) and the mean was 3.09 ± 1.10. Pharmacists had a median of 3.67 (IQR 3.00–4.33) and mean of 3.47 ± 1.02, compared with 2.67 (IQR 2.00–3.33) and 2.70 ± 1.03 among family physicians (Mann–Whitney U = 81,200.5, *p* < 0.001).

The three items were positively intercorrelated (Spearman ρ = 0.62–0.68). Cronbach’s α was 0.847. KMO was 0.729, and Bartlett’s test was significant (χ^2^ = 873.78, df = 3, *p* < 0.001). PCA identified a first component with eigenvalue 2.308 explaining 76.9% of the variance. These findings indicate internal coherence in the development sample only (Figure 2).

IRS ≥ 4 was observed in 185 participants (27.3%), including 136 pharmacists (40.0%) and 49 family physicians (14.5%; χ^2^ = 54.29, df = 1, *p* < 0.001). This threshold is descriptive and was not criterion-validated.

The observed professional-group difference is known-group variation within the development sample, not evidence of construct validity, criterion validity, measurement invariance, reproducibility, or external validity (Table 2 and Table 3, and Figure 3).

### 3.4. Exploratory Logistic Regression and Continuous-Score Sensitivity Analysis

A multivariable logistic regression model explored factors associated with IRS ≥ 4. The model included 678 participants and 185 IRS ≥ 4 cases. It contained 18 explanatory parameters plus an intercept and was prespecified before estimation.

The likelihood-ratio test was significant (*p* < 0.001). Nagelkerke’s pseudo-R^2^ was 0.166 and is reported as an overall model-fit index, not as literal variance explained. The maximum VIF among explanatory terms was 5.32 (age 25–34 years); the remaining VIFs were below 4.25. The maximum Cook’s distance was 0.075, with no observation exceeding 0.10.

Compared with family physicians, pharmacists had higher odds of IRS ≥ 4 (adjusted OR 4.41, 95% CI 2.66–7.33, *p* < 0.001). The crude OR for this comparison was 3.93 (95% CI 2.71–5.71, *p* < 0.001).

Considering direct physician–pharmacist collaboration absolutely necessary was associated with IRS ≥ 4 compared with considering it unnecessary (adjusted OR 4.22, 95% CI 1.42–12.52, *p* = 0.010; crude OR 5.73, 95% CI 2.01–16.31, *p* = 0.001).

Annual personal influenza vaccination was associated with IRS ≥ 4 (adjusted OR 2.98, 95% CI 1.56–5.69, *p* = 0.001), whereas occasional vaccination was not (adjusted OR 1.53, 95% CI 0.81–2.90, *p* = 0.194).

Awareness that influenza vaccination could be provided in community pharmacies was associated with IRS ≥ 4 (adjusted OR 2.06, 95% CI 1.10–3.85, *p* = 0.025). This association should not be interpreted as evidence that awareness caused greater support.

Compared with participants aged ≥55 years, those aged 35–44 years (adjusted OR 2.69, 95% CI 1.16–6.21, *p* = 0.021) and 45–54 years (adjusted OR 2.54, 95% CI 1.37–4.74, *p* = 0.003) had higher odds of IRS ≥ 4. The 25–34-year estimate was imprecise (adjusted OR 2.76, 95% CI 0.94–8.12, *p* = 0.064).

After correction of the category coding, partial current collaboration was not independently associated with IRS ≥ 4 compared with ineffective collaboration (adjusted OR 0.78, 95% CI 0.49–1.25, *p* = 0.298). Effective collaboration was also not independently associated (adjusted OR 1.35, 95% CI 0.81–2.26, *p* = 0.252). The earlier inverse association was therefore not robust to the corrected coding.

Sex and professional experience were not independently associated with IRS ≥ 4 after adjustment. Women had an adjusted OR of 0.70 (95% CI 0.40–1.23, *p* = 0.219) versus men.

Model discrimination was acceptable (AUC 0.775, 95% bootstrap CI 0.734–0.812), and calibration was satisfactory (Hosmer–Lemeshow χ^2^ = 3.87, df = 8, *p* = 0.869).

In the continuous-score sensitivity analysis, the same covariates were entered into a heteroskedasticity-robust linear regression (Figure 4). The principal associations were similar: pharmacist status β = 0.68 (95% CI 0.49–0.87), direct collaboration absolutely necessary β = 1.17 (95% CI 0.85–1.49), annual vaccination β = 0.51 (95% CI 0.28–0.74), and awareness β = 0.24 (95% CI 0.02–0.46). The model R^2^ was 0.308. Full coefficients are provided in the continuous-score sensitivity table below (Table 4 and Table 5).

## 4. Discussion

This study examined professional support for implementing community pharmacy influenza vaccination in Northwestern Romania using a three-item exploratory implementation-support score. The score captures favourable attitudes toward coverage benefit, the pharmacist’s multidisciplinary role, and willingness to recommend pharmacy vaccination; it does not capture the full organisational construct of implementation readiness.

The three items showed internal coherence and a dominant first component in the same sample in which they were selected. These findings support internal consistency only. They do not establish content validity, criterion validity, test–retest reliability, measurement invariance across pharmacists and family physicians, responsiveness, or external validity [14,15,16,17].

Pharmacists had higher IRS values than family physicians. This difference may reflect professional role identification or differential interpretation of items that explicitly concern the pharmacist’s role and recommendation of a pharmacist-delivered service rather than a difference on a common latent readiness construct. Direct comparison across professional groups should therefore remain cautious until measurement invariance is evaluated [18,19,20,21].

Perceiving direct physician–pharmacist collaboration as absolutely necessary was associated with higher IRS values in both the dichotomised and continuous analyses. This pattern is consistent with the practical importance of coordinated pathways, but the cross-sectional design cannot determine directionality [22].

Awareness of pharmacy-based vaccination was associated with greater IRS values. The association may reflect familiarity with the service, professional self-selection, or other unmeasured factors; it should not be interpreted as evidence that awareness increased support [6,23,24,25,26,27,28,29].

Annual personal influenza vaccination was also associated with greater IRS values. This finding is compatible with shared positive attitudes toward vaccination, but self-reported behaviour and support were measured at the same time, and no causal inference is possible [30,31,32].

Age showed exploratory associations with IRS ≥ 4 for the 35–44 and 45–54 year groups compared with participants aged ≥55 years. These estimates should be interpreted cautiously because the analysis was not powered for definitive age-group comparisons, and multiple exploratory contrasts were examined.

The previously reported inverse association for partial collaboration disappeared after corrected category coding. This illustrates the sensitivity of the finding to coding and reference-category decisions and argues against a substantive interpretation of partial collaboration as a barrier in this dataset.

The findings are broadly consistent with the international literature showing that pharmacy vaccination requires training, clear responsibilities, communication pathways, and supportive reimbursement and regulatory conditions [6,13,21,22,23,32,33]. In Romania, the legal framework introduced pharmacy vaccination in 2022, but the present survey did not measure authorisation, training completion, staffing, reimbursement, or workflow capacity at pharmacy level. The data therefore support hypotheses about professional support and collaboration, not a conclusion that organisational readiness itself has been demonstrated.

### 4.1. Practical Implications

The practical implication is that expansion of pharmacy vaccination should be evaluated with multilevel implementation measures rather than with professional attitude items alone. Future work should combine validated measures of organisational readiness with site-level information on authorisation, training, staffing, workflow, reimbursement, documentation, and medico-legal procedures.

Profession-specific education may be useful, but it should be paired with shared clinical pathways, defined responsibilities, interoperable documentation, and mechanisms for physician–pharmacist communication. These are implementation hypotheses requiring prospective evaluation.

The IRS may be useful for hypothesis generation and for planning a future validation study, but it should not currently be used for individual, institutional, or policy decisions. Independent studies should assess content validity, test–retest reliability, factor structure, measurement invariance, criterion or predictive validity, and performance in other regions.

### 4.2. Strengths and Limitations

Strengths include the relatively large and balanced sample, explicit identification of the three score items, and reanalysis using both the exploratory dichotomisation and the continuous score. The study also provides county-level distribution and transparent reporting of the modelling decisions.

The logistic and continuous models allowed for simultaneous evaluation of professional, behavioural, and collaborative correlates. However, no model can remove the limitations of non-probability sampling, self-report, same-sample score construction and evaluation, or possible overlap between IRS components and predictors.

The cross-sectional design precludes causal inference. The overall return rate could not be verified separately by profession because profession-specific distribution denominators were not recorded. Recruitment through pharmacy-linked sites may overrepresent professionals with existing interprofessional contact. The number and selection of participating pharmacies, urban/rural status, site clustering, reimbursement, staffing, training completion, and organisational resources were unavailable. The limited geographic scope also constrains generalisability.

The IRS was created and assessed in the same sample from three attitudinal items that may be vulnerable to professional-group differential item functioning. No independent validation sample, confirmatory factor analysis, test–retest assessment, formal content-validity procedure, criterion validation, or measurement-invariance analysis was available. IRS ≥ 4 was arbitrary and descriptive.

## 5. Conclusions

Healthcare professionals differed in their reported support for community pharmacy influenza vaccination, with higher IRS values among pharmacists than family physicians. The three-item IRS showed internal coherence in the development sample, and its associations with profession, perceived need for direct collaboration, awareness, and annual influenza vaccination were similar in logistic and continuous-score analyses.

These findings should be interpreted as exploratory associations between professional attitudes and a study-specific support index. They do not establish organisational implementation readiness, causality, a validated threshold, measurement invariance, reproducibility, or external validity.

Future implementation programmes should prioritise collaborative clinical pathways, standardised communication, defined responsibilities, training, reimbursement, and supportive regulatory frameworks. Before the IRS is used beyond hypothesis-generating research, the questionnaire and score require independent psychometric and criterion-validation studies.

## Figures and Tables

**Figure 1:**
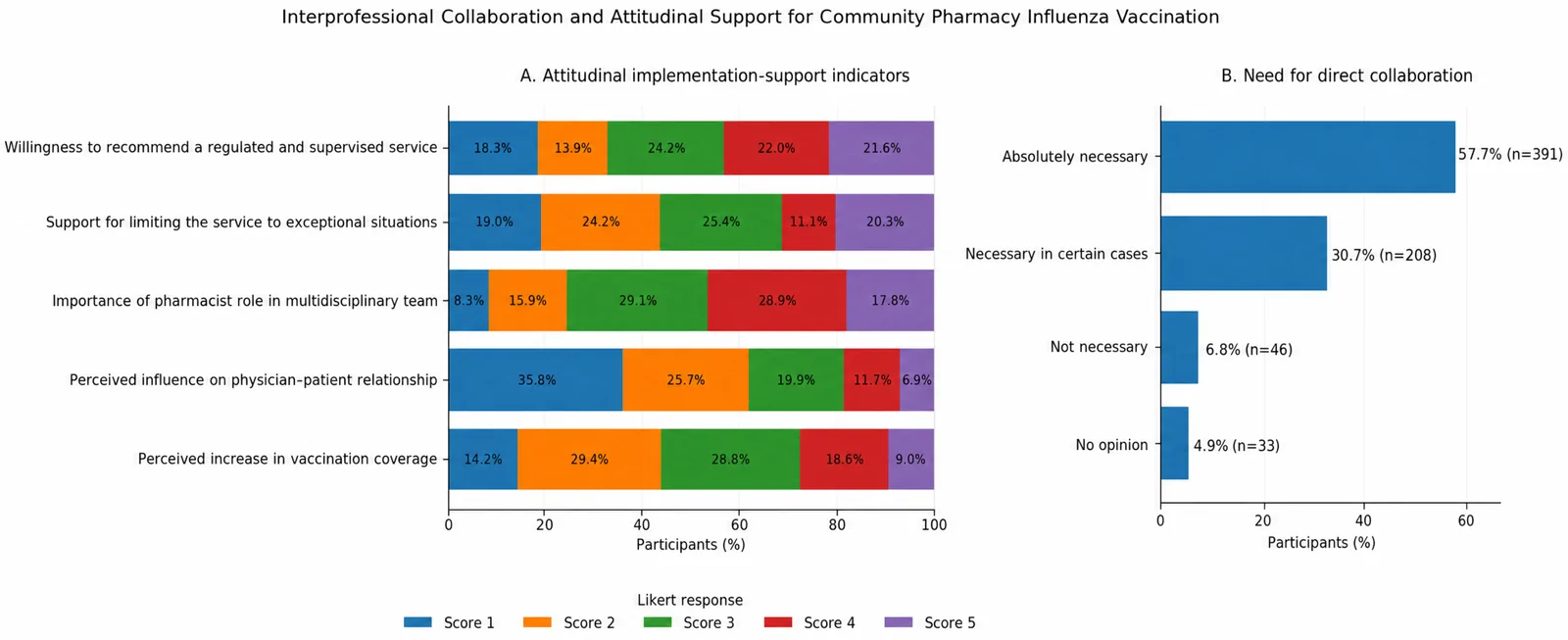
Interprofessional collaboration and attitudinal support for community pharmacy influenza vaccination. Panel (**A**) presents percentage distributions across five-point items for perceived vaccination-coverage benefit, influence on the physician–patient relationship, the pharmacist’s multidisciplinary role, restriction of the service to exceptional situations, and willingness to recommend pharmacy-based vaccination. Panel (**B**) presents perceptions of the need for direct collaboration. Overall, 57.7% considered such collaboration absolutely necessary and 30.7% considered it necessary in selected cases. Percentages may not total exactly 100% because of rounding.

**Figure 2:**
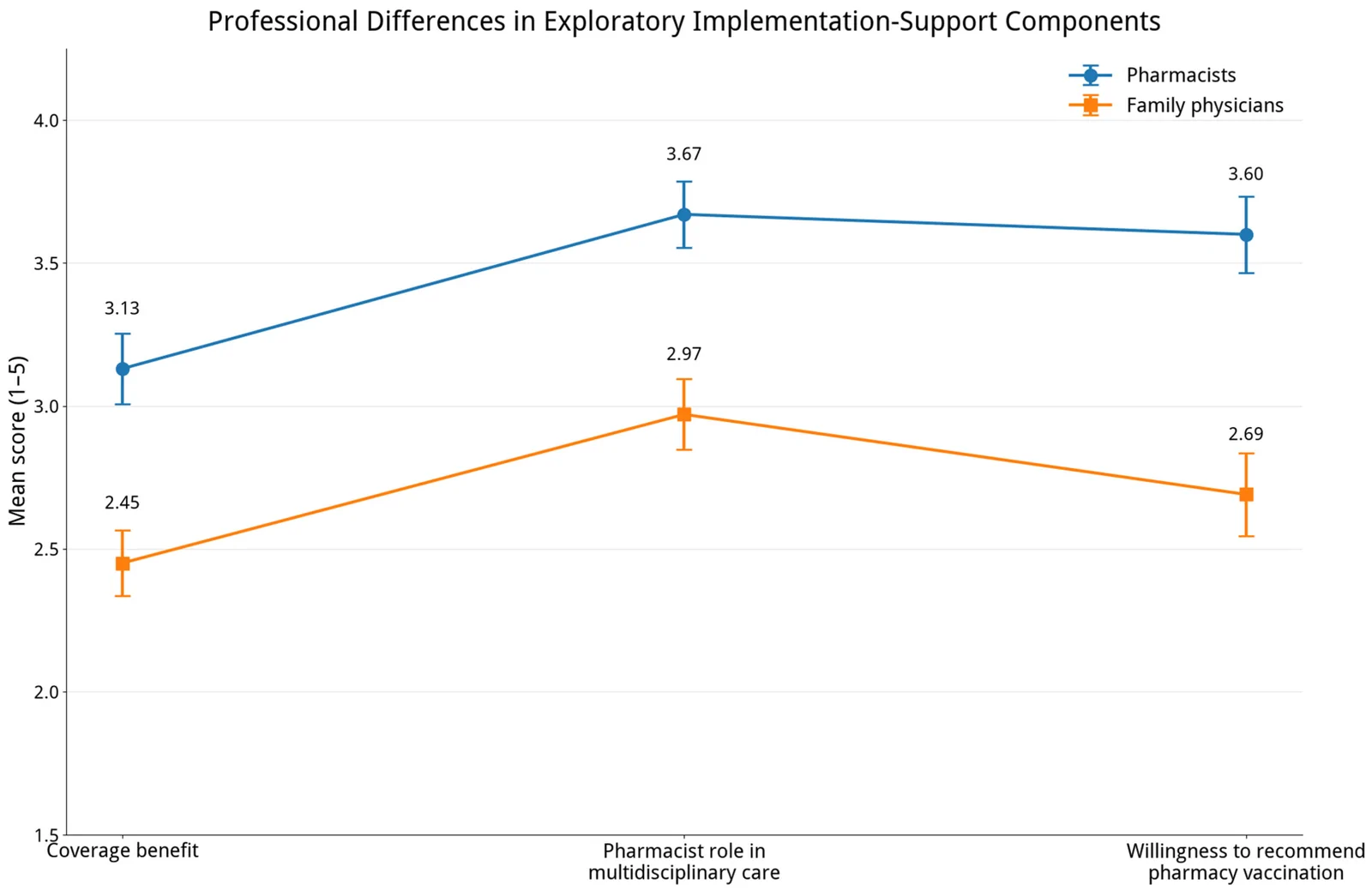
Differences between pharmacists and family physicians in the three exploratory IRS components: perceived vaccination-coverage benefit, the pharmacist’s multidisciplinary role, and willingness to recommend pharmacy-based vaccination. Points represent means and error bars indicate 95% confidence intervals; medians and IQRs are reported in Table 1.

**Figure 3:**
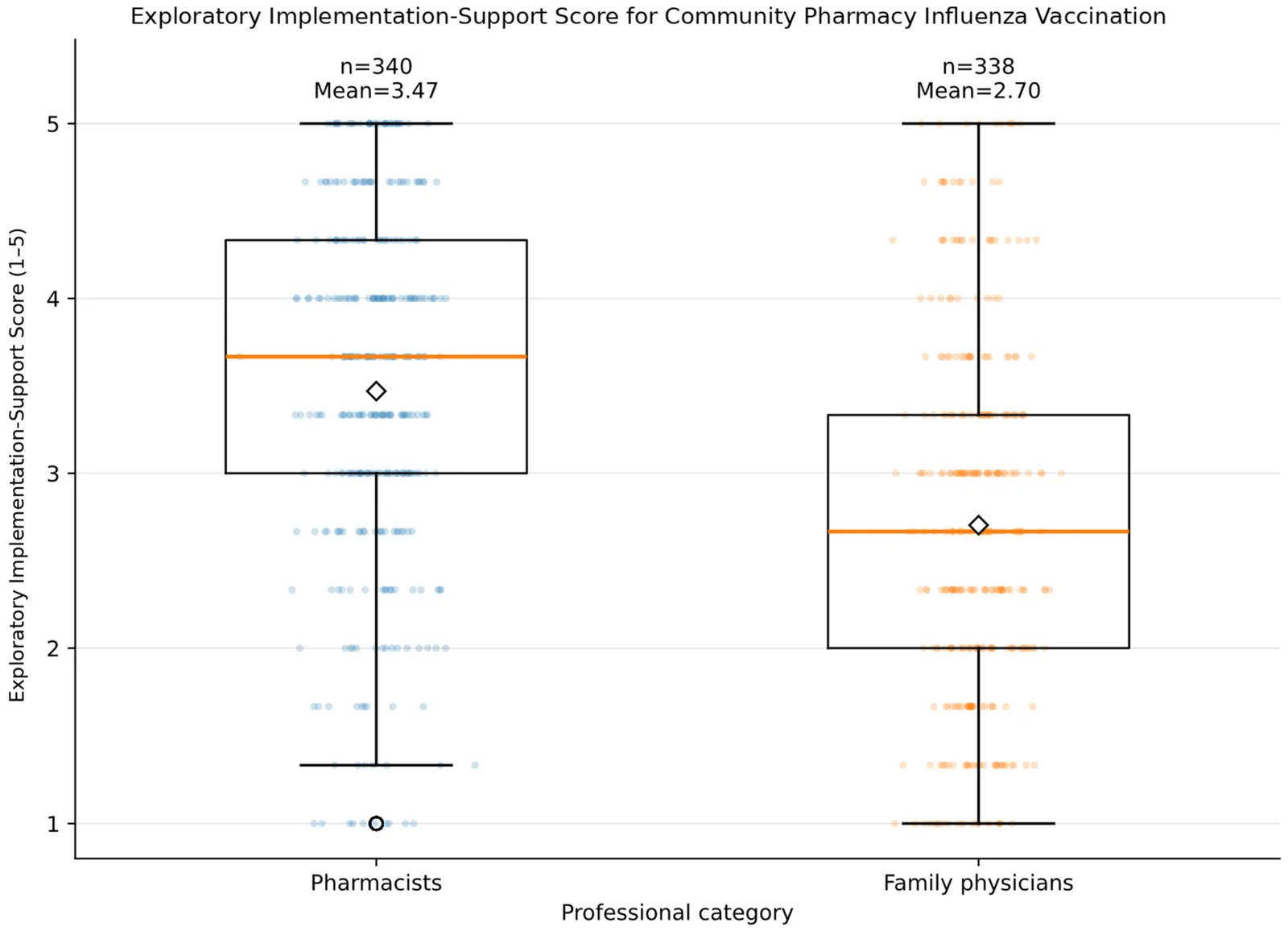
The exploratory implementation-support score (IRS) according to professional category. The observed group difference is descriptive and does not constitute validation of a general implementation-support instrument. Jittered points represent individual responses (blue, pharmacists; orange, family physicians). Boxplots show the interquartile range, with the orange horizontal line indicating the median; whiskers and horizontal caps indicate the non-outlier range, and open circles indicate potential outliers. Black diamonds represent the mean score. The sample sizes were n = 340 for pharmacists and n = 338 for family physicians, with mean scores of 3.47 and 2.70, respectively. Scores ranged from 1 to 5.

**Figure 4:**
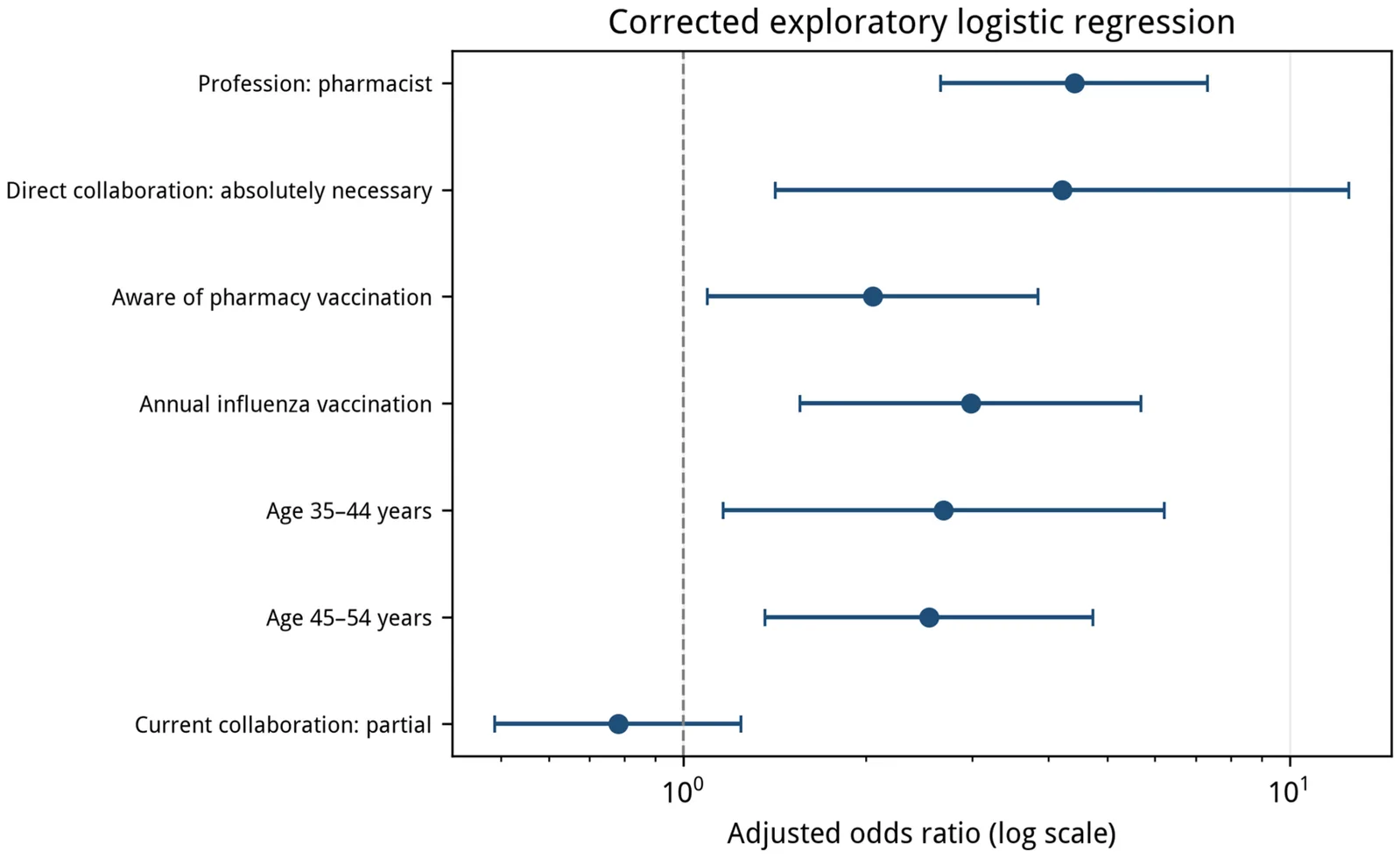
Corrected exploratory logistic regression for IRS ≥ 4. Points represent adjusted odds ratios and horizontal lines indicate 95% confidence intervals. The outcome threshold has not been externally validated.

**Table 1:** Differences between pharmacists and family physicians in interprofessional collaboration and attitudes toward implementation.

Outcome	Pharmacists, Mean ± SD	Pharmacists, Median (IQR)	Family Physicians, Mean ± SD	Family Physicians, Median (IQR)	Mann–Whitney U	*p*-Value
Coverage benefit	3.13 ± 1.16	3 (2–4)	2.45 ± 1.08	2 (2–3)	77,000.5	<0.001
Physician–patient relationship influence	2.40 ± 1.26	2 (1–3)	2.16 ± 1.23	2 (1–3)	64,010.5	0.008
Pharmacist multidisciplinary role	3.67 ± 1.09	4 (3–5)	2.97 ± 1.16	3 (2–4)	76,901.5	<0.001
Restriction to exceptional situations	3.21 ± 1.35	3 (2–5)	2.58 ± 1.35	2 (1–4)	72,312.5	<0.001
Willingness to recommend pharmacy vaccination	3.60 ± 1.26	4 (3–5)	2.69 ± 1.37	3 (1–4)	78,624.5	<0.001

Likert outcomes were compared using the Mann–Whitney U test. Medians and IQRs are the primary descriptive summaries; means and SDs are shown for comparability with prior reports. Higher scores indicate stronger perceived coverage benefit, a more important pharmacist role, or greater willingness to recommend pharmacy vaccination except for the separately analysed restriction item.

**Table 2:** Exploratory implementation-support score (IRS): descriptive and within-sample internal-structure assessment.

Group	*n*	Mean ± SD	Median (IQR)	Minimum–Maximum	IRS ≥ 4, n (%)
Overall	678	3.09 ± 1.10	3.00 (2.33–4.00)	1.00–5.00	185 (27.3)
Pharmacists	340	3.47 ± 1.02	3.67 (3.00–4.33)	1.00–5.00	136 (40.0)
Family physicians	338	2.70 ± 1.03	2.67 (2.00–3.33)	1.00–5.00	49 (14.5)

**Table 3:** Within-sample internal-structure assessment and group comparison.

Cronbach’s α (internal coherence)	0.847
Kaiser–Meyer–Olkin statistic (development sample)	0.729
Bartlett test of sphericity (development sample)	χ^2^ = 873.78, df = 3, *p* < 0.001
First-component eigenvalue	2.308
Variance accounted for by first component	76.9%
Mann–Whitney comparison	U = 81,200.5, *p* < 0.001
Exploratory threshold comparison (IRS ≥ 4)	χ^2^ = 54.29, df = 1, *p* < 0.001

The IRS was the arithmetic mean of the three prespecified attitudinal items. IRS ≥ 4.00 was used only as an exploratory analytical threshold and is not a validated cut-off.

**Table 4:** Multivariable logistic regression analysis of factors associated with IRS ≥ 4.

Predictor Domain	Comparison	Adjusted OR	95% CI Lower	95% CI Upper	*p*-Value
Profession	Pharmacist vs. family physician	4.41	2.66	7.33	<0.001
Current collaboration	Partial vs. ineffective	0.78	0.49	1.25	0.298
Current collaboration	Effective vs. ineffective	1.35	0.81	2.26	0.252
Current collaboration	Not applicable vs. ineffective	1.44	0.42	4.96	0.568
Need for direct collaboration	Selected cases vs. not necessary	1.68	0.54	5.17	0.368
Need for direct collaboration	Absolutely necessary vs. not necessary	4.22	1.42	12.52	0.010
Need for direct collaboration	No opinion vs. not necessary	1.14	0.24	5.38	0.873
Awareness	Aware vs. unaware	2.06	1.10	3.85	0.025
Personal influenza vaccination	Occasional vs. never	1.53	0.81	2.90	0.194
Personal influenza vaccination	Annual vs. never	2.98	1.56	5.69	0.001
Age	45–54 vs. ≥55 years	2.54	1.37	4.74	0.003
Age	35–44 vs. ≥55 years	2.69	1.16	6.21	0.021
Age	25–34 vs. ≥55 years	2.76	0.94	8.12	0.064
Age	<25 vs. ≥55 years	0.22	0.02	2.61	0.231
Sex	Female vs. male	0.70	0.40	1.23	0.219
Professional experience	11–20 vs. >20 years	0.58	0.32	1.06	0.075
Professional experience	5–10 vs. >20 years	0.71	0.30	1.65	0.423
Professional experience	<5 vs. >20 years	1.41	0.52	3.80	0.495

**Table 5:** Model performance.

Participants included	678
IRS ≥ 4 cases	185
Likelihood-ratio test	*p* < 0.001
Nagelkerke pseudo-R^2^	0.166
AUC (95% bootstrap CI)	0.775 (0.734–0.812)
Hosmer–Lemeshow test	χ^2^ = 3.87, df = 8, *p* = 0.869

Outcome: IRS ≥ 4, used as an exploratory dichotomisation rather than a validated cut-off. Adjusted odds ratios were mutually adjusted for professional category, current collaboration, need for direct collaboration, awareness, personal influenza vaccination behaviour, age, sex, and professional experience. Adjusted logistic-regression estimates are reported in Table 4; full coefficients for the continuous-score sensitivity analysis are reported in Supplementary Table S1.

## Data Availability

The original contributions presented in this study are included in this article. Further inquiries can be directed to the first author.

## Supplementary Materials

The following supporting information can be downloaded at https://www.mdpi.com/article/10.3390/epidemiologia7050132/s1, File S1: Verified Romanian questionnaire, author-approved English translation, response anchors, and coding instructions; Table S1: Continuous-score sensitivity analysis using heteroskedasticity-robust linear regression (outcome: IRS, range 1–5).

## Abbreviations

The following abbreviations are used in this manuscript:

Abbreviation	Definition
aOR	Adjusted Odds Ratio
AUC	Area Under the Receiver Operating Characteristic Curve
CI	Confidence Interval
IQR	Interquartile Range
OR	Odds Ratio
ROC	Receiver Operating Characteristic
SD	Standard Deviation

## References

[1] Kyokha Ameen Y. (2025). Seasonal Influenza: A Narrative Review of Epidemiology, Clinical Features, and Preventive Strategies. Cureus.

[2] Rzymski P. (2026). Potential Non-Specific Benefits of Seasonal Influenza Vaccination: Evidence, Knowledge Gaps, and Future Directions. Vaccines.

[3] Waite N.M., Houle S.K.D., Toppari K., Pereira J.A. (2024). Willingness of Canadian community pharmacists to adopt a proactive life-course approach to vaccination services. J. Am. Pharm. Assoc..

[4] Valeiro C., Silva V., Balteiro J., Patterson D., Bezerra G., Mealiff K., Matos C., Jesus Â., Joaquim J. (2025). Pharmacy Technicians in Immunization Services: Mapping Roles and Responsibilities Through a Scoping Review. Healthcare.

[5] G/Michael Beyene K., Tamiru M.T. (2025). Quality and safety requirements for pharmacy-based vaccination in resource-limited countries. Trop. Med. Health.

[6] Chereches M.C., Naidin M.S., Grosan A., Patrascu R.A., Capraru A.-M., Dimulescu M.D., Turcu-Stiolica A. (2026). Pharmacist-Led Flu Vaccination Services in Romanian Community Pharmacies: Barriers, Perceptions, and Implementation Challenges. Pharmacy.

[7] Bouton C., Journeaux M., Jourdain M., Angibaud M., Huon J.F., Rat C. (2023). Interprofessional collaboration in primary care: What effect on patient health? A systematic literature review. BMC Prim. Care.

[8] Haems M., Lanzilotto M., Mandelli A., Mota-Filipe H., Paulino E., Plewka B., Rozaire O., Zeiger J. (2024). European community pharmacists practice in tackling influenza. Explor. Res. Clin. Soc. Pharm..

[9] AlMahasis S.O., Fox B., Ha D., Qian J., Wang C.H., Westrick S.C. (2024). Rural community pharmacists’ willingness to receive, administer, and recommend COVID-19 vaccines after the First U.S. vaccine rollout. Explor. Res. Clin. Soc. Pharm..

[10] Udoh A., Ernawati D., Ikhile I., Yahyouche A. (2024). Pharmacists’ Willingness to Offer Vaccination Services: A Systematic Review and Meta-Analysis. Pharmacy.

[11] Livet M., Blanchard C., Richard C. (2022). Readiness as a precursor of early implementation outcomes: An exploratory study in specialty clinics. Implement Sci. Commun..

[12] Chirila S., Hangan T., Gurgas L., Costache M.G., Vlad M.A., Nitu B.F., Bittar S.M., Craciun A., Condur L., Bjørklund G. (2024). Pharmacy-Based Influenza Vaccination: A Study of Patient Acceptance in Romania. Risk Manag. Healthc. Policy.

[13] Buicu C.-F., Naidin M.-S., Chereches M.C., Dimulescu M.-D., Turcu-Stiolica A. (2025). Pharmacists’ perceptions of the new pharmaceutical vaccination service in Romania: A comprehensive first two-years evaluation. Front. Pharmacol..

[14] Rahim M.H.A., Dom S.H.M., Hamzah M.S.R., Azman S.H., Zaharuddin Z., Fahrni M.L. (2024). Impact of pharmacist interventions on immunisation uptake: A systematic review and meta-analysis. J. Pharm. Policy Pract..

[15] Elghanam Y., Kim E.Y. (2025). Impact of pharmacist intervention on enhancing vaccination coverage: A systematic review and meta-analysis. Res. Soc. Adm. Pharm..

[16] Farrell B., Pottie K., Woodend K., Yao V., Dolovich L., Kennie N., Sellors C. (2010). Shifts in expectations: Evaluating physicians’ perceptions as pharmacists become integrated into family practice. J. Interprof. Care.

[17] Clemens S., Eisl-Raudaschl L., Pachmayr J., Rose O. (2025). Community Pharmacist Prescribing: Roles and Competencies—A Systematic Review and Implications. Pharmacy.

[18] Ramsey N.C., Peterson G.M., Mirkazemi C., Salahudeen M.S. (2025). Factors influencing medical Prescribers’ Acceptance of Pharmacists’ recommendations in non-hospitalized older adults: A systematic review and meta-analysis. J. Am. Med. Dir. Assoc..

[19] Côté L., Normandeau M., Maheux B., Authier L., Lefort L. (2013). Collaboration between family physicians and community pharmacists: Opinions of graduates in family medicine. Can. Fam. Physician.

[20] Wrześniewska-Wal I., Pinkas J., Ostrowski J., Jankowski M. (2023). Pharmacists’ Perceptions of Physician–Pharmacist Collaboration—A 2022 Cross-Sectional Survey in Poland. Healthcare.

[21] Thomas D., Abdalla A., Hussein S., Joury J., Elshamy A., Khalifa S., Saleh Z. (2023). Pharmacists’ readiness and willingness to vaccinate the public in United Arab Emirates community pharmacies: A cross-sectional study. F1000Research.

[22] Ayenew W., Anagaw Y.K., Limenh L.W., Simegn W., Bizuneh G.K., Bitew T., Minwagaw T., Fitigu A.E., Dessie M.G., Asmamaw G. (2024). Readiness of and barriers for community pharmacy professionals in providing and implementing vaccination services. BMC Health Serv. Res..

[23] Papastergiou J., Folkins C., Li W., Zervas J. (2014). Community pharmacist-administered influenza immunization improves patient access to vaccination. Can. Pharm. J..

[24] Al-Hamdan Z., Al Hamdan M., Bawadi H., Al Hamdan A., Alzoubi M.M., Al Mugheed K., Saeed Alabdullah A.A., Farghaly Abdelaliem S.M. (2026). Motivators and barriers to influenza vaccination among healthcare workers. Hum. Vaccin Immunother..

[25] Kougioumtzoglou I., Maniadakis N., Kouvelas D., Kostaki E.-G., Selekos N., Koulouvari A.-D., Lagiou A. (2026). Beliefs, Attitudes and Behaviors of Healthcare Professionals Regarding Seasonal Influenza Vaccination: An Umbrella Review. Germs.

[26] Guillari A., Polito F., Pucciarelli G., Serra N., Gargiulo G., Esposito M.R., Botti S., Rea T., Simeone S. (2021). Influenza vaccination and healthcare workers: Barriers and predisposing factors. Acta Biomed..

[27] Veronese N., Dominguez L.J., Ganci A., Speziale G., Mansueto P., Piro S., Basile G., Barbagallo M. (2025). Influenza vaccination in older people: A geriatrician’s perspective. Aging Clin. Exp. Res..

[28] Du N., Shao S., Zhang Y., Liu C., Pang Y., Hang H., Chen L. (2026). Influenza Vaccination Status and Associated Factors Among Older Adults in Suzhou, China: A Comparative Cross-Sectional Survey. Vaccines.

[29] Morgan S., Pullon S., McKinlay E. (2026). Observation of interprofessional collaborative practice in primary care teams: An updated integrative review. Int. J. Nurs. Stud..

[30] Green B.N., Johnson C.D. (2015). Interprofessional collaboration in research, education, and clinical practice: Working together for a better future. J. Chiropr. Educ..

[31] Jaam M., Naseralallah L.M., Hussain T.A., Pawluk S.A. (2021). Pharmacist-led educational interventions provided to healthcare providers to reduce medication errors: A systematic review and meta-analysis. PLoS ONE.

[32] Ministry of Health of Romania Order no. 3262/19 October 2022 for the Approval of the Organization and Operation of a Pilot Program for Seasonal Influenza Vaccination at Community Pharmacies. https://legislatie.just.ro/Public/DetaliiDocumentAfis/260720.

[33] World Health Organization Regional Office for Europe (2022). Increasing the Accessibility of Influenza Vaccination in Romania. https://eurohealthobservatory.who.int/monitors/health-systems-monitor/updates/hspm/romania-2016/increasing-the-accessibility-of-the-influenza-vaccination.

