# Supplementary material for: Interprofessional Collaboration and Exploratory Implementation Support for Community Pharmacy Influenza Vaccination in Northwestern Romania: A Cross-Sectional Survey of Pharmacists and Family Physicians
Source: Epidemiologia (Basel). 2026 Sep 22;7(5):132. doi: 10.3390/epidemiologia7050132 (PMC13703594; doi:10.3390/epidemiologia7050132)
Supplement: Supplementary file 1 [file epidemiologia-07-00132-s001.zip › File S1.pdf]

Supplementary File S1

Verified Romanian questionnaire, author-approved English translation, response anchors, and coding instructions

Interprofessional Collaboration and Exploratory Implementation Support for Community Pharmacy Influenza Vaccination in Northwestern Romania: A Cross-Sectional Survey of Pharmacists and Family Physicians

Source and verification. The Romanian instrument below is the final questionnaire supplied and verified by the lead author. The accompanying English translation has been reviewed and approved by the authors for publication. Only typographical, spelling, and spacing inconsistencies in the working Romanian file were standardised; item meaning, response options, and item order were not changed.

General coding conventions. Blank responses are coded as missing (NA) and are not imputed. For multiple-response items, each option is stored as a separate binary indicator (1 = selected; 0 = not selected). Free-text responses are stored verbatim. “Do not know”, “no opinion”, and “not applicable” responses remain distinct categorical values and are not interpreted as positions on an ordinal scale.

Complete bilingual instrument and item-level coding

Item	Original Romanian instrument	Author-approved English translation	Response format and coding
**I. Date sociodemografice / I. Sociodemographic data**			
1	**Profesie:**<br>☐ Medic de familie<br>☐ Farmacist	**Profession:**<br>☐ Family physician<br>☐ Pharmacist	Single choice.<br>1 = family physician; 2 = pharmacist.<br>Regression reference: family physician.
2	**Câți ani de experiență aveți în profesia dumneavoastră?**<br>☐ <5 ani<br>☐ 5–10 ani<br>☐ 11–20 ani<br>☐ >20 ani	**How many years of professional experience do you have?**<br>☐ <5 years<br>☐ 5–10 years<br>☐ 11–20 years<br>☐ >20 years	Single choice.<br>1 = <5; 2 = 5–10; 3 = 11–20; 4 = >20 years.
3	**Județul din zona de Nord-Vest în care vă desfășurați activitatea ca profesionist în domeniul sănătății:**<br>☐ Bihor<br>☐ Bistrița-Năsăud<br>☐ Cluj<br>☐ Maramureș<br>☐ Satu Mare<br>☐ Sălaj	**County in the Northwestern region where you practise as a healthcare professional:**<br>☐ Bihor<br>☐ Bistrița-Năsăud<br>☐ Cluj<br>☐ Maramureș<br>☐ Satu Mare<br>☐ Sălaj	Single choice.<br>1 = Bihor; 2 = Bistrița-Năsăud; 3 = Cluj; 4 = Maramureș; 5 = Satu Mare; 6 = Sălaj.

Item	Original Romanian instrument	Author-approved English translation	Response format and coding
4	**Ce vârstă aveți?**<br>☐ <25 ani<br>☐ 25–34 ani<br>☐ 35–44 ani<br>☐ 45–54 ani<br>☐ ≥55 ani	**What is your age?**<br>☐ <25 years<br>☐ 25–34 years<br>☐ 35–44 years<br>☐ 45–54 years<br>☐ ≥55 years	Single choice.<br>1 = <25; 2 = 25–34; 3 = 35–44; 4 = 45–54; 5 = ≥55 years.
5	**Genul:**<br>☐ Feminin<br>☐ Masculin	**Sex:**<br>☐ Female<br>☐ Male	Single choice.<br>1 = female; 2 = male. Regression reference: male.
**II. Cunoștințe și atitudini / II. Knowledge and attitudes**			
6	**Care este importanța pe care o acordați vaccinării antigripale pentru sănătate?**<br>1 = Deloc important<br>2 = Puțin important<br>3 = Moderat<br>4 = Important<br>5 = Foarte important	**How important do you consider influenza vaccination for health?**<br>1 = Not at all important<br>2 = Slightly important<br>3 = Moderately important<br>4 = Important<br>5 = Very important	Single choice; ordinal 1–5. Higher scores indicate greater perceived importance.
7	**Care sunt principalele surse de informare pe care le utilizați în ceea ce privește vaccinarea? (puteți selecta mai multe răspunsuri)**<br>☐ Medicul de familie<br>☐ Farmacist<br>☐ Literatură medicală de specialitate<br>☐ Internet<br>☐ Mass-media (ziare, radio, TV)<br>☐ Persoane apropiate (membri ai familiei, vecini, prieteni)<br>☐ Alte surse: _________	**What are the main sources of information you use regarding vaccination? (select all that apply)**<br>☐ Family physician<br>☐ Pharmacist<br>☐ Specialist medical literature<br>☐ Internet<br>☐ Mass media (newspapers, radio, TV)<br>☐ Close contacts (family members, neighbours, friends)<br>☐ Other sources: _________	Multiple response. Each option: 1 = selected, 0 = not selected. “Other” text stored verbatim.
8	**Considerați vaccinarea o strategie eficientă de prevenire a bolilor?**<br>1 = Da<br>2 = Parțial<br>3 = Nu<br>4 = Nu știu	**Do you consider vaccination an effective disease-prevention strategy?**<br>1 = Yes<br>2 = Partly<br>3 = No<br>4 = Do not know	Single choice; categorical. Codes follow the displayed response order.
9	**Sunteți la curent cu posibilitatea vaccinării în farmacii?**<br>1 = Da<br>0 = Nu	**Are you aware that vaccination can be provided in pharmacies?**<br>1 = Yes<br>0 = No	Single choice; binary.<br>1 = aware; 0 = unaware.

Item	Original Romanian instrument	Author-approved English translation	Response format and coding
**10**	**În ce măsură ați fi dispus(ă) să vă vaccinați într-o farmacie?**<br>1 = Deloc dispus(ă)<br>2 = Puțin dispus(ă)<br>3 = Indecis(ă)<br>4 = Dispus(ă)<br>5 = Foarte dispus(ă)	**How willing would you be to receive a vaccination in a pharmacy?**<br>1 = Not at all willing<br>2 = Slightly willing<br>3 = Undecided<br>4 = Willing<br>5 = Very willing	Single choice; ordinal 1–5. Higher scores indicate greater willingness.
**III. Percepții și experiențe / III. Perceptions and experiences**			
**11**	**Ați beneficiat personal de vaccinarea antigripală? Dacă da, cât de des vă vaccinați?**<br>0 = Nu m-am vaccinat niciodată<br>1 = Vaccinare ocazională<br>2 = Vaccinare anuală	**Have you personally received influenza vaccination? If yes, how often are you vaccinated?**<br>0 = Never vaccinated<br>1 = Occasionally vaccinated<br>2 = Vaccinated annually	Single choice; categorical.<br>0 = never; 1 = occasional; 2 = annual.
**12**	**Aveți încredere că autoritățile publice iau decizii potrivite legate de vaccinarea antigripală, având în vedere binele întregii comunități?**<br>1 = Deloc<br>2 = Puțin<br>3 = Moderat<br>4 = Mult<br>5 = Foarte mult	**Do you trust public authorities to make appropriate influenza-vaccination decisions in the interest of the whole community?**<br>1 = Not at all<br>2 = To a small extent<br>3 = To a moderate extent<br>4 = To a great extent<br>5 = To a very great extent	Single choice; ordinal 1–5. Higher scores indicate greater trust.
**13**	**Cât de importantă considerați că este încrederea în siguranța și eficacitatea vaccinurilor, precum și în sistemul de sănătate?**<br>1 = Deloc important/irelevant<br>2 = Puțin important<br>3 = Moderat important<br>4 = Important<br>5 = Foarte important	**How important do you consider trust in vaccine safety and effectiveness, as well as trust in the healthcare system?**<br>1 = Not at all important/irrelevant<br>2 = Slightly important<br>3 = Moderately important<br>4 = Important<br>5 = Very important	Single choice; ordinal 1–5. Higher scores indicate greater perceived importance.
**14**	**Vaccinarea dumneavoastră este benefică pentru sănătatea celorlalți membri ai comunității?**<br>1 = Da<br>2 = Nu<br>3 = Nu știu	**Is your own vaccination beneficial to the health of other members of the community?**<br>1 = Yes<br>2 = No<br>3 = Do not know	Single choice; categorical. “Do not know” is retained as a distinct category.
**15**	**Ați utilizat vreodată serviciile de vaccinare oferite în farmacii?**<br>1 = Da<br>0 = Nu	**Have you ever used vaccination services provided in pharmacies?**<br>1 = Yes<br>0 = No	Single choice; binary.<br>1 = yes; 0 = no.
**16**	**Dacă da, cum evaluați experiența?**<br>1 = Deloc satisfăcător<br>2 = Puțin satisfăcător<br>3 = Moderat satisfăcător<br>4 = Satisfăcător<br>5 = Foarte satisfăcător	**If yes, how do you rate the experience?**<br>1 = Not at all satisfactory<br>2 = Slightly satisfactory<br>3 = Moderately satisfactory<br>4 = Satisfactory<br>5 = Very satisfactory	Single choice; ordinal 1–5. Applicable only when Item 15 = 1; otherwise coded missing/not applicable.

Item	Original Romanian instrument	Author-approved English translation	Response format and coding
17	**Care considerați că sunt principalele beneficii ale vaccinării în farmacii?**<br>☐ Creșterea accesului populației la vaccinuri<br>☐ Decongestionarea cabinetelor de medicină de familie<br>☐ Creșterea aderenței la vaccinare<br>☐ Timp de așteptare redus<br>☐ Flexibilitate a programului	**What do you consider the main benefits of vaccination in pharmacies?**<br>☐ Increased public access to vaccines<br>☐ Reduced workload in family-medicine practices<br>☐ Increased adherence to vaccination<br>☐ Reduced waiting time<br>☐ Flexible opening hours	Multiple response. Each option: 1 = selected, 0 = not selected.
18	**Cum apreciați nivelul de încredere al pacienților în farmacist în calitate de furnizor de servicii de vaccinare?**<br>1 = Deloc încrezători<br>2 = Puțin încrezători<br>3 = Moderat încrezători<br>4 = Încrezători<br>5 = Foarte încrezători	**How do you rate patients' level of trust in pharmacists as providers of vaccination services?**<br>1 = Not at all trusting<br>2 = Slightly trusting<br>3 = Moderately trusting<br>4 = Trusting<br>5 = Highly trusting	Single choice; ordinal 1–5. Higher scores indicate greater perceived trust.
19	**Considerați că posibilele reacții adverse ale vaccinurilor afectează în mod semnificativ balanța risc–beneficiu?**<br>1 = Deloc<br>2 = Puțin<br>3 = Moderat<br>4 = Mult<br>5 = Foarte mult	**Do you consider that possible vaccine adverse reactions significantly affect the risk–benefit balance?**<br>1 = Not at all<br>2 = To a small extent<br>3 = To a moderate extent<br>4 = To a great extent<br>5 = To a very great extent	Single choice; ordinal 1–5. Higher scores indicate a greater perceived effect.
**IV. Bariere și soluții / IV. Barriers and solutions**			
20	**Care considerați că sunt principalele temeri ale pacienților legate de vaccinarea în farmacii? (puteți selecta mai multe răspunsuri)**<br>☐ Lipsa siguranței/eficienței vaccinului<br>☐ Lipsa personalului medical pregătit pentru reacții adverse<br>☐ Costul vaccinului<br>☐ Lipsa informațiilor suficiente<br>☐ Alte temeri: ______________	**What do you consider the main patient concerns regarding vaccination in pharmacies? (select all that apply)**<br>☐ Concerns about vaccine safety/effectiveness<br>☐ Lack of medical personnel trained to manage adverse reactions<br>☐ Vaccine cost<br>☐ Insufficient information<br>☐ Other concerns: ______________	Multiple response. Each option: 1 = selected, 0 = not selected. "Other" text stored verbatim.
21	**Ce temeri considerați că pot avea profesioniștii din sănătate în legătură cu vaccinarea în farmacii? (puteți selecta mai multe răspunsuri)**<br>☐ Lipsa de pregătire adecvată a personalului farmacist<br>☐ Absența condițiilor optime de siguranță (spații, echipamente)<br>☐ Posibile reacții adverse și gestionarea lor<br>☐ Responsabilitate medico-legală neclară<br>☐ Alte temeri: __________	**What concerns do you think healthcare professionals may have regarding vaccination in pharmacies? (select all that apply)**<br>☐ Inadequate training of pharmacy staff<br>☐ Lack of optimal safety conditions (premises, equipment)<br>☐ Possible adverse reactions and their management<br>☐ Unclear medico-legal responsibility<br>☐ Other concerns: __________	Multiple response. Each option: 1 = selected, 0 = not selected. "Other" text stored verbatim.

Item	Original Romanian instrument	Author-approved English translation	Response format and coding
22	**În opinia dumneavoastră, cum este percepută acceptarea vaccinurilor de către populație în prezent în țara noastră?**<br>1 = Percepție foarte negativă / respingere ridicată<br>2 = Percepție negativă / respingere moderată<br>3 = Percepție neutră / acceptare moderată<br>4 = Percepție pozitivă / acceptare ridicată<br>5 = Percepție foarte pozitivă / acceptare foarte ridicată	**In your opinion, how is vaccine acceptance currently perceived among the population in Romania?**<br>1 = Very negative perception / high rejection<br>2 = Negative perception / moderate rejection<br>3 = Neutral perception / moderate acceptance<br>4 = Positive perception / high acceptance<br>5 = Very positive perception / very high acceptance	Single choice; ordinal 1–5. Higher scores indicate more positive perceived acceptance.
23	**Cum apreciați gradul de acceptabilitate al vaccinării în farmacii în rândul profesioniștilor din domeniul sănătății din județul dumneavoastră?**<br>1 = Foarte acceptabil<br>2 = Acceptabil<br>3 = Moderat<br>4 = Puțin acceptabil<br>5 = Inacceptabil	**How do you rate the acceptability of vaccination in pharmacies among healthcare professionals in your county?**<br>1 = Very acceptable<br>2 = Acceptable<br>3 = Moderate<br>4 = Slightly acceptable<br>5 = Unacceptable	Single choice; ordinal 1–5; codes follow the displayed order. Higher scores indicate lower acceptability.
24	**Cât de preocupat(ă) sunteți de riscul de malpraxis în contextul administrării vaccinurilor?**<br>1 = Deloc preocupat(ă)<br>2 = Puțin preocupat(ă)<br>3 = Moderat preocupat(ă)<br>4 = Preocupat(ă)<br>5 = Foarte preocupat(ă)	**How concerned are you about malpractice risk in the context of vaccine administration?**<br>1 = Not at all concerned<br>2 = Slightly concerned<br>3 = Moderately concerned<br>4 = Concerned<br>5 = Very concerned	Single choice; ordinal 1–5. Higher scores indicate greater concern.
25	**Cine considerați că ar trebui să fie responsabil legal pentru eventualele reacții adverse ale vaccinării efectuate în farmacie?**<br>1 = Farmacistul<br>2 = Medicul coordonator<br>3 = Casa de Asigurări<br>4 = Ministerul Sănătății	**Who do you consider should be legally responsible for possible adverse reactions following vaccination performed in a pharmacy?**<br>1 = Pharmacist<br>2 = Supervising physician<br>3 = Health Insurance House<br>4 = Ministry of Health	Single choice; nominal categorical. Codes follow the displayed response order.
26	**În ce măsură administrarea vaccinurilor se potrivește competențelor farmacistului?**<br>1 = Deloc potrivită<br>2 = Mai degrabă nepotrivită<br>3 = Nici potrivită, nici nepotrivită<br>4 = Mai degrabă potrivită<br>5 = Foarte potrivită	**To what extent does vaccine administration fit within pharmacists' competencies?**<br>1 = Not at all suitable<br>2 = Rather unsuitable<br>3 = Neither suitable nor unsuitable<br>4 = Rather suitable<br>5 = Very suitable	Single choice; ordinal 1–5. Higher scores indicate greater perceived fit.
27	**Farmacistul nu este pregătit să ofere vaccinare în condiții sigure.**<br>1 = Complet de acord<br>2 = De acord<br>3 = Nici de acord, nici în dezacord<br>4 = În dezacord<br>5 = Complet în dezacord	**The pharmacist is not prepared to provide vaccination safely.**<br>1 = Strongly agree<br>2 = Agree<br>3 = Neither agree nor disagree<br>4 = Disagree<br>5 = Strongly disagree	Single choice; ordinal 1–5. Negatively worded item; raw codes follow the displayed order. Not included in the IRS.

Item	Original Romanian instrument	Author-approved English translation	Response format and coding
28	**În ce măsură ați recomanda vaccinarea antigripală în farmacii altor persoane?**<br>1 = Cu siguranță nu aș recomanda<br>2 = Probabil nu aș recomanda<br>3 = Nu sunt sigur(ă)<br>4 = Probabil aș recomanda<br>5 = Cu siguranță aș recomanda	**To what extent would you recommend influenza vaccination in pharmacies to other people?**<br>1 = Definitely would not recommend<br>2 = Probably would not recommend<br>3 = Not sure<br>4 = Probably would recommend<br>5 = Definitely would recommend	Single choice; ordinal 1–5. Higher scores indicate greater willingness to recommend.
29	**Ce condiții considerați esențiale pentru a permite farmacistului să administreze vaccinuri?**<br>☐ Pregătire și formare<br>☐ Spațiu și echipamente corespunzătoare<br>☐ Supraveghere medicală<br>☐ Protocoale de siguranță<br>☐ Integrarea în sistemul de sănătate publică	**What conditions do you consider essential for allowing pharmacists to administer vaccines?**<br>☐ Education and training<br>☐ Appropriate premises and equipment<br>☐ Medical supervision<br>☐ Safety protocols<br>☐ Integration into the public healthcare system	Multiple response. Each option: 1 = selected, 0 = not selected.
30	**Cât de important considerați ca toți profesioniștii din domeniul sănătății să recomande pacienților vaccinarea?**<br>1 = Deloc important<br>2 = Puțin important<br>3 = Moderat important<br>4 = Important<br>5 = Foarte important	**How important is it for all healthcare professionals to recommend vaccination to patients?**<br>1 = Not at all important<br>2 = Slightly important<br>3 = Moderately important<br>4 = Important<br>5 = Very important	Single choice; ordinal 1–5. Higher scores indicate greater perceived importance.
31	**Care considerați că reprezintă principalele bariere în implementarea serviciului de vaccinare antigripală în județul dumneavoastră? (puteți selecta mai multe răspunsuri)**<br>☐ Bariere psihologice (superstiții și mituri legate de vaccinare)<br>☐ Bariere organizaționale (lipsa personalului și a materialelor necesare)<br>☐ Bariere economice (costul serviciului de vaccinare)<br>☐ Bariere legislative/administrative (proceduri birocratice complicate)<br>☐ Bariere sociale (influența negativă a grupurilor anti-vaccinare)	**What do you consider the main barriers to implementing influenza-vaccination services in your county? (select all that apply)**<br>☐ Psychological barriers (superstitions and vaccination myths)<br>☐ Organisational barriers (insufficient staff and materials)<br>☐ Economic barriers (cost of the vaccination service)<br>☐ Legislative/administrative barriers (complex bureaucratic procedures)<br>☐ Social barriers (negative influence of anti-vaccination groups)	Multiple response. Each option: 1 = selected, 0 = not selected.
32	**Pe baza experienței proprii, în ce măsură considerați că barierele și constrângerile afectează procesul decizional legat de imunizare?**<br>1 = Deloc<br>2 = Puțin<br>3 = Moderat<br>4 = Mult<br>5 = Foarte mult	**Based on your own experience, to what extent do barriers and constraints affect immunisation-related decision-making?**<br>1 = Not at all<br>2 = To a small extent<br>3 = To a moderate extent<br>4 = To a great extent<br>5 = To a very great extent	Single choice; ordinal 1–5. Higher scores indicate a greater perceived effect.
**V. Colaborare interdisciplinară și perspective / V. Interdisciplinary collaboration and perspectives**			

Item	Original Romanian instrument	Author-approved English translation	Response format and coding
33	**Care considerați că este locul preferat de către pacienți pentru efectuarea vaccinării antigripale?**<br>1 = La medicul de familie<br>2 = La farmacie<br>3 = La spital<br>4 = Nu știu	**Where do you think patients prefer to receive influenza vaccination?**<br>1 = Family physician's office<br>2 = Pharmacy<br>3 = Hospital<br>4 = Do not know	Single choice; nominal categorical. "Do not know" is retained as a distinct category.
34	**Considerați că administrarea vaccinului antigripal în farmacii ar putea crește acoperirea vaccinală în județul dumneavoastră?**<br>1 = Deloc<br>2 = Puțin<br>3 = Moderat<br>4 = Mult<br>5 = Foarte mult	**To what extent do you consider that administering the influenza vaccine in pharmacies could increase vaccination coverage in your county?**<br>1 = Not at all<br>2 = To a small extent<br>3 = To a moderate extent<br>4 = To a great extent<br>5 = To a very great extent	Single choice; ordinal 1–5. Higher scores indicate greater perceived coverage benefit. IRS Item 1.
35	**În ce măsură considerați că vaccinarea antigripală administrată în farmacii ar putea influența relația medic–pacient?**<br>1 = Deloc<br>2 = Puțin<br>3 = Moderat<br>4 = Mult<br>5 = Foarte mult	**To what extent do you consider that influenza vaccination administered in pharmacies could influence the physician–patient relationship?**<br>1 = Not at all<br>2 = To a small extent<br>3 = To a moderate extent<br>4 = To a great extent<br>5 = To a very great extent	Single choice; ordinal 1–5. Higher scores indicate a greater perceived influence; direction is not inherently favourable. Not included in the IRS.
36	**Cum vedeți rolul farmacistului în echipa multidisciplinară implicată în vaccinare?**<br>1 = Deloc important<br>2 = Puțin important<br>3 = Moderat<br>4 = Important<br>5 = Foarte important	**How do you view the pharmacist's role in the multidisciplinary team involved in vaccination?**<br>1 = Not at all important<br>2 = Slightly important<br>3 = Moderately important<br>4 = Important<br>5 = Very important	Single choice; ordinal 1–5. Higher scores indicate a more important perceived role. IRS Item 2.
37	**Considerați că există o colaborare eficientă între medicii de familie și farmaciști în privința campaniilor de vaccinare din județul dumneavoastră?**<br>3 = Da<br>2 = Parțial<br>1 = Nu<br>0 = Nu este cazul în zona mea	**Do you consider that family physicians and pharmacists collaborate effectively on vaccination campaigns in your county?**<br>3 = Yes<br>2 = Partly<br>1 = No<br>0 = Not applicable in my area	Single choice; categorical.<br>0 = not applicable; 1 = ineffective/no; 2 = partial; 3 = effective/yes.
38	**Considerați că este necesară o colaborare directă între medic și farmacist în procesul de vaccinare?**<br>1 = Da, absolut necesară<br>2 = Da, în anumite cazuri<br>3 = Nu este necesară<br>4 = Nu am o opinie	**Do you consider direct collaboration between the physician and pharmacist necessary in the vaccination process?**<br>1 = Yes, absolutely necessary<br>2 = Yes, in selected cases<br>3 = Not necessary<br>4 = No opinion	Single choice; categorical; codes follow the displayed order. Regression reference: not necessary. "No opinion" remains separate.

Item	Original Romanian instrument	Author-approved English translation	Response format and coding
39	**Serviciul de vaccinare în farmacii ar trebui limitat exclusiv la situații excepționale?**<br>1 = Deloc limitat<br>2 = Puțin limitat<br>3 = Moderat limitat<br>4 = Limitat<br>5 = Foarte limitat	**Should vaccination services in pharmacies be restricted exclusively to exceptional situations?**<br>1 = Not restricted at all<br>2 = Slightly restricted<br>3 = Moderately restricted<br>4 = Restricted<br>5 = Highly restricted	Single choice; ordinal 1–5. Higher scores indicate stronger support for restriction. Not included in the IRS.
40	**În ce măsură ați fi dispus(ă) să recomandați pacienților vaccinarea antigripală în farmacii, cu condiția ca serviciul să fie reglementat și supravegheat corespunzător?**<br>1 = Deloc<br>2 = Puțin<br>3 = Moderat<br>4 = Mult<br>5 = Foarte mult	**To what extent would you be willing to recommend influenza vaccination in pharmacies to patients, provided that the service is appropriately regulated and supervised?**<br>1 = Not at all<br>2 = To a small extent<br>3 = To a moderate extent<br>4 = To a great extent<br>5 = To a very great extent	Single choice; ordinal 1–5. Higher scores indicate greater willingness to recommend. IRS Item 3.
41	**În ultima perioadă, cât de des ați avut cereri din partea pacienților pentru servicii de vaccinare în farmacie?**<br>1 = Da, frecvent – serviciile de vaccinare sunt foarte căutate în farmacie<br>2 = Da, ocazional – există un interes moderat pentru vaccinare<br>3 = Foarte rar – cererile pentru vaccinare sunt sporadice<br>4 = Niciodată – pacienții nu solicită vaccinarea în farmacie<br>5 = Nu știu/nu răspund	**Recently, how often have patients requested vaccination services in the pharmacy?**<br>1 = Frequently – vaccination services are in high demand<br>2 = Occasionally – there is moderate interest<br>3 = Very rarely – requests are sporadic<br>4 = Never – patients do not request pharmacy vaccination<br>5 = Do not know/prefer not to answer	Single choice; categorical codes 1–5 follow the displayed response order. Do not treat code 5 as an ordinal frequency value.
42	**Ce măsuri considerați că ar putea crește acceptabilitatea serviciului de vaccinare în farmacii?**<br>☐ Campanii de informare și educare a pacienților privind siguranța și beneficiile vaccinării<br>☐ Formarea continuă a farmaciștilor pentru administrarea vaccinurilor<br>☐ Crearea unor protocoale clare de colaborare între medicii de familie și farmaciști<br>☐ Comunicarea clară privind gestionarea reacțiilor adverse<br>☐ Promovarea serviciului prin canale media sau materiale informative în farmacie<br>☐ Alte sugestii: ___________	**What measures do you consider could increase the acceptability of vaccination services in pharmacies?**<br>☐ Patient information and education campaigns on vaccination safety and benefits<br>☐ Continuing training for pharmacists in vaccine administration<br>☐ Clear collaboration protocols between family physicians and pharmacists<br>☐ Clear communication on adverse-reaction management<br>☐ Promotion through media channels or informational materials in pharmacies<br>☐ Other suggestions: ___________	Multiple response. Each option: 1 = selected, 0 = not selected. "Other" text stored verbatim.
**VI. Sugestii finale / VI. Final suggestions**			
43	**Ce schimbări sau îmbunătățiri ați propune pentru serviciul de vaccinare în farmacii?**<br>Răspuns deschis:<br>______________________________	**What changes or improvements would you propose for vaccination services in pharmacies?**<br>Open response:<br>______________________________	Free text stored verbatim. Not included in the quantitative analyses reported in the manuscript.

IRS derivation and analysis-specific coding

Element	Definition	Coding/analysis rule

Element	Definition	Coding/analysis rule
**IRS Item 1**	Item 34: perceived contribution to vaccination coverage	Raw score 1–5; higher = greater support.
**IRS Item 2**	Item 36: pharmacist's role in the multidisciplinary team	Raw score 1–5; higher = greater support.
**IRS Item 3**	Item 40: willingness to recommend a regulated and supervised service	Raw score 1–5; higher = greater support.
**IRS score**	Arithmetic mean of Items 34, 36, and 40	All three responses required; theoretical range 1–5. No reverse-coded IRS item.
**Exploratory threshold**	IRS ≥4.00	Descriptive analytical dichotomisation only; not a validated clinical, institutional, or policy cut-off.
**Continuous sensitivity outcome**	IRS retained as a continuous score	Analysed using heteroskedasticity-robust linear regression.
