# Supplementary material for: Interprofessional Collaboration and Exploratory Implementation Support for Community Pharmacy Influenza Vaccination in Northwestern Romania: A Cross-Sectional Survey of Pharmacists and Family Physicians
Source: Epidemiologia (Basel). 2026 Sep 22;7(5):132. doi: 10.3390/epidemiologia7050132 (PMC13703594; doi:10.3390/epidemiologia7050132)
Supplement: Supplementary file 1 [file epidemiologia-07-00132-s001.zip › Table S1.pdf]

Supplementary Table S1. Continuous-score sensitivity analysis using heteroskedasticity-robust linear regression (outcome: IRS, range 1–5).

Predictor	β	95% CI lower	95% CI upper	p-value
Pharmacist vs. family physician	0.68	0.49	0.87	<0.001
Current collaboration: effective vs. ineffective	0.25	0.05	0.45	0.013
Current collaboration: partial vs. ineffective	0.02	-0.16	0.19	0.846
Direct collaboration: selected cases vs. not necessary	0.77	0.44	1.10	<0.001
Direct collaboration: absolutely necessary vs. not necessary	1.17	0.85	1.49	<0.001
Aware vs. unaware	0.24	0.02	0.46	0.029
Annual vaccination vs. never	0.51	0.28	0.74	<0.001
Occasional vaccination vs. never	0.27	0.04	0.50	0.021
Age 35–44 vs. ≥55 years	0.59	0.29	0.88	<0.001
Age 45–54 vs. ≥55 years	0.54	0.32	0.75	<0.001
Female vs. male	-0.19	-0.40	0.02	0.070
Experience 11–20 vs. >20 years	-0.29	-0.52	-0.06	0.013

The continuous-score model used the same reference categories and covariates as the logistic model. Coefficients represent the adjusted mean difference in the IRS.
